# Trace Amine-Associated Receptor 5 Provides Olfactory Input Into Limbic Brain Areas and Modulates Emotional Behaviors and Serotonin Transmission

**DOI:** 10.3389/fnmol.2020.00018

**Published:** 2020-03-05

**Authors:** Stefano Espinoza, Ilya Sukhanov, Evgeniya V. Efimova, Alena Kozlova, Kristina A. Antonova, Placido Illiano, Damiana Leo, Natalia Merkulyeva, Daria Kalinina, Pavel Musienko, Anna Rocchi, Liudmila Mus, Tatiana D. Sotnikova, Raul R. Gainetdinov

**Affiliations:** ^1^Department of Neuroscience and Brain Technologies, Istituto Italiano di Tecnologia, Genova, Italy; ^2^Department of Pharmacology, St. Petersburg State Medical University, St. Petersburg, Russia; ^3^Institute of Translational Biomedicine, St. Petersburg State University, St. Petersburg, Russia; ^4^Pavlov Institute of Physiology RAS, St. Petersburg, Russia; ^5^Sechenov Institute of Evolutionary Physiology and Biochemistry RAS, St. Petersburg, Russia; ^6^St. Petersburg State Research Institute of Phthisiopulmonology, Ministry of Healthcare of the RF, St. Petersburg, Russia; ^7^Center for Synaptic Neuroscience and Technology, Istituto Italiano di Tecnologia, Genoa, Italy; ^8^IRCSS Ospedale Policlinico San Martino, Genoa, Italy; ^9^St. Petersburg State University Hospital, St. Petersburg State University, St. Petersburg, Russia

**Keywords:** TAAR5, TAAR, trace amine, depression, anxiety, olfaction, antidepressant, anxiolytic

## Abstract

Trace amine-associated receptors (TAARs) are a class of G-protein-coupled receptors found in mammals. While TAAR1 is expressed in several brain regions, all the other TAARs have been described mainly in the olfactory epithelium and the glomerular layer of the olfactory bulb and are believed to serve as a new class of olfactory receptors sensing innate odors. However, there is evidence that TAAR5 could play a role also in the central nervous system. In this study, we characterized a mouse line lacking TAAR5 (TAAR5 knockout, TAAR5-KO) expressing beta-galactosidase mapping TAAR5 expression. We found that TAAR5 is expressed not only in the glomerular layer in the olfactory bulb but also in deeper layers projecting to the limbic brain olfactory circuitry with prominent expression in numerous limbic brain regions, such as the anterior olfactory nucleus, the olfactory tubercle, the orbitofrontal cortex (OFC), the amygdala, the hippocampus, the piriform cortex, the entorhinal cortex, the nucleus accumbens, and the thalamic and hypothalamic nuclei. TAAR5-KO mice did not show gross developmental abnormalities but demonstrated less anxiety- and depressive-like behavior in several behavioral tests. TAAR5-KO mice also showed significant decreases in the tissue levels of serotonin and its metabolite in several brain areas and were more sensitive to the hypothermic action of serotonin 5-HT1A receptor agonist 8-hydroxy-2-(di-*n*-propilamino)tetralin (8-OH-DPAT). These observations indicate that TAAR5 is not just innate odor-sensing olfactory receptor but also serves to provide olfactory input into limbic brain areas to regulate emotional behaviors likely *via* modulation of the serotonin system. Thus, anxiolytic and/or antidepressant action of future TAAR5 antagonists could be predicted. In general, “olfactory” TAAR-mediated brain circuitry may represent a previously unappreciated neurotransmitter system involved in the transmission of innate odors into emotional behavioral responses.

## Introduction

Trace amine-associated receptors (TAARs) are a family of G-protein-coupled receptors (GPCRs) discovered in 2001 and expressed in vertebrates (Borowsky et al., 2001; Bunzow et al., 2001). Although the numbers of TAAR genes differ between species, they can be classified into nine subfamilies, with different numbers of pseudogenes present in every species (Gainetdinov et al., 2018). While TAAR1 expression has been reported in the brain as well as in peripheral organs, the other TAARs appear to be expressed in the olfactory epithelium of all the species studied (Liberles, 2015). However, there is evidence suggesting the expression of different TAARs outside the olfactory system (Berry et al., 2017). Before the discovery of TAARs family, TAAR5 was initially known as putative neurotransmitter receptor (PNR), and expression of TAAR5 messenger RNA (mRNA) was reported in the human amygdala (Zeng et al., 1998). More recently, TAAR5 mRNA was found in several mouse brain areas, including the amygdala, the arcuate nucleus, and the hypothalamus (Dinter et al., 2015). Moreover, TAAR5 transcripts were detected also in peripheral organs, such as testis, intestines, and leukocytes (Berry et al., 2017). As for other TAARs, the main problem for mapping the expression of these receptors is the low abundancy of the transcripts, the limit of detection of the techniques used, and the small areas where these transcripts are present. TAAR5 can be activated by tertiary amines, especially by trimethylamine (TMA), that act as full agonist at TAAR5 (Liberles and Buck, 2006). TMA is present in mouse urine and is attractive in mice, while it is repulsive in rats and humans, and this effect is mediated by TAAR5 (Li et al., 2013). Several weak TAAR5 antagonists have been recently identified, with the most notable antagonistic action of an amber-woody fragrance Timberol (Wallrabenstein et al., 2015; Cichero et al., 2016). Furthermore, an inverse agonistic action of 3-iodothyronamine has been also reported (Dinter et al., 2015). However, outside the olfactory system, whether TAAR5 is expressed in the brain and which physiological functions could have it is still a matter of debate. In our study, we take advantage of a mouse line lacking TAAR5 (TAAR5 knockout mice, TAAR5-KO) that express the beta-galactosidase enzyme under the control of TAAR5 promoter. With this reporter mouse line, we detected TAAR5 expression not only in the olfactory system but also in discrete limbic brain regions that receive input from the olfactory bulb. Moreover, using a series of behavioral tests in mutant mice, we found that the lack of TAAR5 altered emotional behavior, in particular anxiety-and depression-like behavior, and modulated brain serotonin (5-HT) neurotransmission.

## Materials and Methods

### Generation of TAAR-KO Mice

The TAAR5-KO mice were generated by Deltagen Incorporation (San Mateo, CA, USA) by methods previously described (Hall et al., 2009) and distributed by the NIH Knockout Mouse Project (KOMP^1^). Briefly, the target region (from 1 to 320) of trace amine-associated receptor 5 (Taar5; NCBI Gene ID: 215854; mRNA NCBI Ref Seq: NM_001009574; XM_136992) was inactivated through a replacement vector by homologous recombination. The targeting vector was assembled from this genomic fragment with a 200 bp flanking the Neo insert, a LacZ-coding sequence fused to a nuclear localization sequence (NLS), a PgK-NeoR, and a diphtheria toxin cassette. The targeting vector was linearized with SacII and electroporated into C57BL/6 embryonic stem (ES) cells, and G-418 (Geneticin; Invitrogen, Paisley, UK)—resistant ES cell clones were selected. An ES cell clone carrying a homologous recombination event was identified by PCR, and it was used to generate chimeras according to standard protocols. The recombinant allele was maintained in C57BL/6 background in a specific pathogen-free facility with a 12:12-h day/night cycle and *ad libitum* access to food and water. The correct homologous recombination was further confirmed by PCR amplification of fragment PCR from genomic DNA and by Southern blot to confirm the deletion of the target gene. For genotyping, DNA was isolated from mice tails in overnight incubation in a tail lysis buffer [0.1 M Tris–HCl, 0.005 M ethylenediaminetetraacetic acid (EDTA), 0.2% sodium dodecyl sulfate, 0.5 mg/ml proteinase K] at +55°C. Proteinase K was then deactivated by heating the sample at +95°C, followed by adding 0.02 mg/ml RNase A in Tris–EDTA buffer (1 M Tris–HCl, 0.1 M EDTA, pH 7.4). Relying on the insertion of the LacZ gene into the TAAR5 mice line, polymerase chain reaction (PCR) was based on three primers (forward primers: TAG AGC AGG GGG TCA CAG ATG GCA C; GGG GAT CGA TCC GTC CTG TAA GTC T; reverse primer: TGT AGA CAG GGT GAC CAG TTC CCA G). This mixture was used to detect fragments of 480 or 600 bp length, or both, which detect TAAR5+/+, TAAR5−/−, and TAAR5+/− genotypes, respectively. The PCR amplification reaction was performed as follows: 2 min at 95°C, 35 cycles of 30 s at 95°C, 30 s at 60°C, and 60 s at 72°C, and a final elongation step for 5 min at 72°C. The reaction volume was 25 μl and contained 1× DreamTaq Green buffer, 0.14 μM primers, 0.2 mM deoxynucleoside triphosphates (dNTPs), 1.2 mM MgCl_2_, and 0.625 U DreamTaq polymerase (EP0705, Thermo Fisher Scientific, Waltham, MA, USA). PCR products were run on a 2% agarose gel stained with 0.2 mg/ml of ethidium bromide.

### Animals

All procedures involving animals and their care were carried out in accordance with the guidelines established by the European Community Council (Directive 2010/63/EU of September 22, 2010) and were approved by the Italian Ministry of Health and Ethics Committee of St. Petersburg State University, St. Petersburg, Russia. C57Bl/6 littermate (3–5 months old) wild-type (WT) and TAAR5-KO mice were used in the experiments (*n* = 6–22 per group; for details on the number and gender of animals used in each experimental protocol, please see Supplementary Table S1). In the circular open field test, light-dark transition test, elevated plus maze test, and for serotonin measurements by high-performance liquid chromatography (HPLC), only male mice were used. In all other experiments, mice of both sexes were tested. In these experiments, male and female mice were analyzed separately, and since no significant gender differences were found, the data were combined. The mice were housed three to five per cage and maintained under standard lab conditions (12 h light/dark cycle, 21 ± 1°C and 40–70% humidity), with food and water provided *ad libitum*. All experiments were conducted during the light phase, and the same types of experiments were conducted at the same short time period of the day. One hour before behavioral experiments, the mice were habituated to an experimental room.

### Histochemistry

The mice were pre-anesthetized with isoflurane and anesthetized with urethane at a dose of 2 g/kg. The animals were transcardially perfused with 20 ml/min of ice-cold saline (0.9% *w*/*v*) for 5 min and fixative [2% (*w*/*v*) paraformaldehyde and 0.2% (*w*/*v*) glutaraldehyde in phosphate-buffered saline (PBS), pH 7.4]. Brains were postfixed for 4 h in fixative at 4°C, and cryoprotected overnight in 0.5 M sucrose in 0.1 M PBS. Tissue sections were cut on a cryostat (Leica) at 30 μm, thaw mounted on gelatin-coated glass slides (Thermo Fisher Scientific, Waltham, MA, USA), and air dried at room temperature for 4–6 h and processed immediately for LacZ staining. For LacZ staining, tissue sections were washed five times for 10 min in PBS and then incubated for 18 h in LacZ-staining solution [1 mg/ml 5-bromo-4-chloro-3-indolyl-D-galactopyranoside, 5 mM K_3_Fe(CN)_6_, 5 mM K_4_Fe(CN)_6_, and 2 mM MgCl_2_ in PBS] at 37°C. The staining was stopped by washing the tissue sections five times for 10 min at room temperature in PBS. The tissue sections were dehydrated through an ascending ethanol series, equilibrated to xylene, coverslipped with dibutylphthalate polystyrene xylene (DPX; Sigma–Aldrich, St. Louis, MO, USA), and analyzed on an Olympus BX51 Neurolucida microscope.

### Behavioral Tests

#### General Health, Sensory Reflexes, and Motor Abilities

The mouse was weighed, its body temperature was taken, and the appearance of its fur and whiskers was noted. Neurological reflexes were tested in each mouse. These included eye blink, ear twitch, righting reflex, whisker-orienting reflex, and wire hang test. Eye blink reflex was tested by approaching the eye with the tip of a clean cotton swab, ear twitch reflex was tested by touching the ear with the tip of a clean cotton swab, righting reflex was evaluated by turning the mouse onto its back, and whisker-orienting reflex was tested by lightly brushing the whiskers of a freely moving animal with a small paint brush. In the wire hang test, the mouse was placed on a wire cage lid, and the lid was gently waved in the air so the mouse grips the wire. The lid was then turned upside down, approximately six in above the surface of a soft bedding material. Latency to fall onto the bedding was recorded, with a 60-s cut off time.

#### Locomotor Activity

Locomotor activity of the mice was evaluated using an automated Omnitech Digiscan apparatus (AccuScan Instruments, Columbus, OH, USA) under illuminated conditions. The apparatus included four locomotor activity monitors [40 cm (L) × 40 cm (W) × 40 cm (H)], each of which consisted a set of 16 light beams arrayed in the horizontal *X*- and *Y*-axes. The cages were divided into four compartments [20 cm (L) × 20 cm (W)]. The hardware detected beams broken by the animal, which allowed the software to determine the location of the mouse in the cage. Animals were tested individually for defined periods with 5-min intervals.

#### Circular Open Field Test

Circular open field test was used to measure locomotor and exploratory activity. The apparatus consisted of gray plastic round arena (diameter, 67 cm) with 13 holes in the arena floor (hole diameter, 1 cm). The mice were placed at the center of the arena, and spontaneous exploration activity was recorded with the Noldus Ethovision videotracking software for 7 min. The following parameters were scored: total distance moved, velocity, cumulative duration in the central zone and frequency in the central zone, total time of grooming, number of rearings, and number of holes explorations.

#### Light–Dark Transition Test

The apparatus used for the light–dark transition test consisted of a cage [40 cm (L) × 20 cm (W) × 20 cm (H)] divided into two sections of equal size by a partition with a hole. One chamber was brightly illuminated by white diodes, whereas the other chamber was dark. The mice were placed into the dark side. The mice were allowed to move freely between the two chambers, and their activity was recorded for 5 min. The total number of transitions, the total number of nose pokes into the light chamber, the time spent in each chamber, and the latency to enter into the light chamber were quantified.

#### Elevated Zero-Maze Test

The elevated zero maze (Med Associates, St. Albans, VT, USA) consisted of an elevated (60 cm) circular platform (55 cm inner diameter; 60 cm outside diameter), equally divided into four quadrants and elevated above the floor: two quadrants on opposite sides were enclosed by walls, and the other two quadrants were open. The mouse was placed at one closed arm entrance and allowed to move freely for 5 min. The maze was monitored by an overhead mounted camera. The percentage of time spent in the open arms was calculated.

#### Elevated Plus Maze Test

For measuring the level of anxiety-like behavior, we used the elevated plus maze test. Maze consisted of two opposite open (30 × 10 cm) and two opposite enclosed arms (30 × 10 × 15 cm), elevated 30 cm from the floor. Each mouse was placed at the center of the elevated plus maze facing the open arm and was allowed 5 min for free exploration. The following parameters were registered using the Noldus Ethovision software: the cumulative duration in open arms, the frequency in open arms, total distance moved separately in opened and closed arms, velocity, the number of rearing, and the number of head dipping.

#### Learned Helplessness Test

The test was performed as described (Newton et al., 2002) in four apparatus (TSE system, Hamburg, Germany). On day 1, 120 scrambled, randomized inescapable shocks (15 s duration, 0.45 mA, every 45 ± 15 s) were delivered to the mice of stressed group placed individually in one of apparatus compartment. On day 2, two-way avoidance training was initiated. This test session consisted of 30 trials in which electric footshock (0.45 mA, 24 s duration), at random intervals (mean of 30 s; averaging 22–38 s), was preceded by a 3-s conditioned stimulus tone that remained on until the shock was terminated. The animals could transfer to another compartment during condition signal (avoidance) or change a compartment after the footshock starting. The number of avoidances was scored.

#### Temperature Measurement

Core temperature was determined using a digital thermometer (BIO-TK8851, BIOSEB, France). The probe was inserted into the rectum by lightly restraining the animal for ~2.5 cm and maintained until the temperature reading stabilized. To evaluate the effects of drugs, rectal temperatures were assessed every 10 min. The assessments started 20 min before drug administration and went on 60 min after the injection.

### HPLC Measurements of the Tissue Content of Serotonin and its Metabolite

HPLC measurements of tissue 5-HT and its metabolite 5-hydroxyindoleacetic acid (5-HIAA) were carried out as described before Belov et al. (2019). Briefly, the striatum, the frontal cortex, the hypothalamus, and the hippocampus were dissected on an ice, frozen in a liquid nitrogen, and stored at −80°C. The samples for analysis were homogenized in 0.1 M HClO_4_, centrifuged (10 min, +4°C; 14,000× *g*) and filtered using centrifuge filter units [polyvinylidene fluoride (PVDF) membrane; pore size, 0.22 μm, Millipore, Burlington, MA, USA]. Measurements of 5-HT and 5-HIAA in the tissue samples were performed using HPLC with electrochemical detection (Eicom, HTEC-500, Japan) with a carbon electrode WE-3G (Eicom, Japan) using +650 mV applied potential. The system was equipped with a reverse-phase column CA-50DS (150 × 2.1 mm, Eicom, Japan) at a flowrate of 200 μl/min. The mobile phase contained 100 mM sodium-phosphate buffer, 0.17 mM EDTA, 1.8 mM octanesulfonic acid sodium salt, and 18% (vol/vol) methanol, pH 4.5. All peaks obtained were normalized to internal standard 3,4-dihydroxybenzylamine, and final values for 5-HT and 5-HIAA were expressed as nanogram per milligram wet tissue weight. The serotonin turnover rate was calculated as a ratio of 5-HIAA/5-HT.

### Statistical Analysis

Data were analyzed by two-tailed Student’s *t*-test, one-way ANOVA, or two-way ANOVA with Bonferroni *post hoc* test. Values in graphs were expressed as means ± SEM. HPLC measurement data were analyzed using nonparametrical Mann–Whitney *U* test.

## Results

### Generation of TAAR5-KO Mice

**Figure 1:**
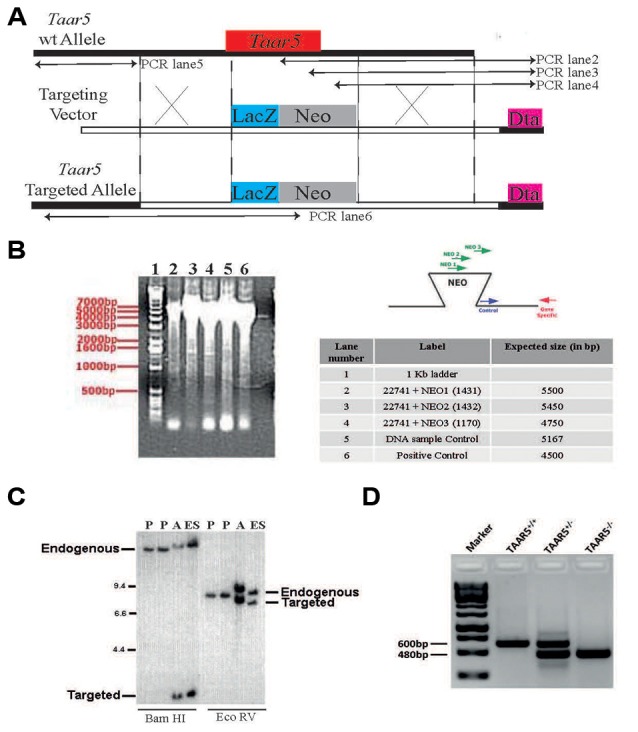
Generation of TAAR5 knockout (TAAR5-KO) mice. **(A)** TAAR5-KO mice were generated using homologous recombination that produces TAAR5 gene inactivation through a replacement vector. The target gene is aligned with the targeting vector so that exon 1 (from base 1 to 320, see “Material and Methods” section) is substituted with the NEOr gene (HSV-tk, negative selection marker). **(B)** Genomic DNA from the recombinant embryonic stem (ES) line was assayed for homologous recombination using PCR. Amplified DNA fragments were visualized by ethidium bromide staining following agarose gel electrophoresis, as shown in the gel image on the left. The test PCRs (a set of three lanes) employed the gene-specific (GS) primer, which lies outside of and beside the targeting vector arm, paired in succession with one of three primers in the insertion fragment. The “DNA sample control” employed a primer pair intended to amplify a fragment from a nontargeted genomic locus. The “positive control” employed the GS primer paired with a primer at the other end of the arm. The table lists the primers (numbered) used in each PCR and the expected product sizes, in base pairs (bp). The diagrams to the right depict the anticipated gel image as well as the relative positions of the PCR primers. **(C)** Genomic DNA isolated from the indicated ES lines was digested with the indicated restriction enzymes (determined to cut outside of the construct arms). The DNA was analyzed by Southern hybridization, probing with a radiolabeled DNA fragment that hybridizes outside of and beside the construct arm. The parent ES lines (negative controls) showed bands representing the endogenous (wild-type) gene #336 allele. In contrast, the ES#3232 line showed an additional band representing the targeted allele from the expected homologous recombination event. “Additional” denotes a secondary line that was derived from the same targeting vector but was not used to generate mice. **(D)** Standard genotyping PCR with genomic DNA of TAAR5-WT (wild type), TAAR5-HET (heterozygous), and TAAR5-KO (knockout) mice.

To address the physiological role of TAAR5 *in vivo*, a targeted mouse mutant was generated in which the bases from 1 to 320 of the Taar5 coding sequence were replaced by a reporter gene consisting of LacZ fused to an NLS (Figure 1A). Homologous recombination in ES cells and homozygous mutants were detected by PCR (Figure 1B). The gene replacement was further confirmed by Southern blot (Figure 1C). Genotyping of mutant mice was performed by PCR as shown (Figure 1D) and described in “Materials and Methods” section.

### TAAR5 Is Expressed in the Olfactory Bulb and Several Limbic Brain Regions

**Figure 2:**
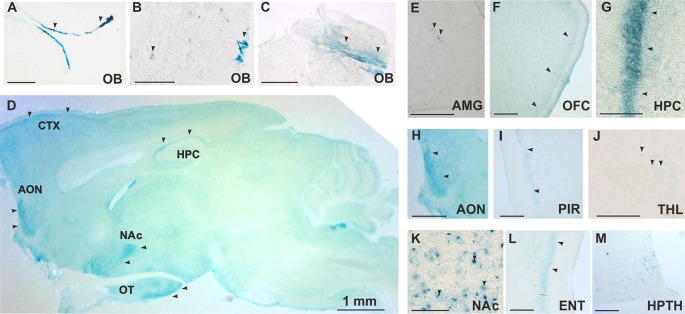
Histological analysis of TAAR5 expression in TAAR5-KO mouse brain by LacZ staining. **(A,B,E–M)** Coronal sections, **(C,D)** Sagittal sections. (**A–C**) Staining in glomerular **(A,B)** and mitral **(B,C)** layers of the olfactory bulb (OB). **(D)** Overview of TAAR5+ regions on sagittal section of whole mouse brain (AON, anterior olfactory nucleus; CTX, cortex; HPC, hippocampus; NAc, nucleus accumbens; OT, olfactory tubercle); **(E)** single TAAR5+ cells in the amygdala (AMG); **(F)** TAAR5+ cells in the orbitofrontal cortex (OFC); **(G)** staining in CA1 region of the hippocampus (HPC); **(H)** TAAR5+ staining in the anterior olfactory nucleus (AON); **(I)** TAAR5+ staining in the piriform cortex (PIR); **(J)** single TAAR5+ cells in the thalamus (THL); **(K)** TAAR5+ cells in the nucleus accumbens (NAc); **(L)** TAAR5+ staining in the entorhinal cortex (ENT); **(M)** TAAR5+ cells in the ventromedial hypothalamus (HPTH). Scale bar: (**A–C**) 250 μM, **(D)** 1 mm, **(E,G,K,M)** 100 μM, **(F,H,I,J,L)** 500 μM. Arrows indicate LacZ staining.

TAAR5 expression in the brain was analyzed by the use of the *LacZ* reporter gene that was inserted in frame in place of the *Taar5* gene, a single exon gene. By analyzing a series of stained brain sections of KO animals, we noted a discrete and specific LacZ labeling of some brain regions. Staining was performed on sagittal and coronal sections. The strongest staining was found in the olfactory bulb in both of the glomerular (Figures 2A,B) and mitral (Figures 2B,C) layers. TAAR5 was expressed also in the several brain regions as shown in the brain sagittal section from TAAR5-KO mouse (Figure 2D). Localized expression was detected in the amygdala (Figure 2E) and the orbitofrontal cortex (OFC; Figure 2F). High levels of TAAR5 expression was shown also in the CA1 area of the hippocampus (Figure 2G), anterior olfactory nucleus (Figure 2H), and the piriform cortex (Figure 2I). LacZ detection was also observed in the thalamic region (Figure 2J), the nucleus accumbens (Figure 2K), the entorhinal cortex (Figure 2L), and the ventromedial nucleus of the hypothalamus (Figure 2M). Both male and female mice were assessed in these experiments, and no difference in LacZ signals between genders was noted. Furthermore, we did not observe any unspecific staining in these areas in WT animals (data not shown).

### General Behavioral Profile of TAAR5-KO Mice

**Figure 3:**
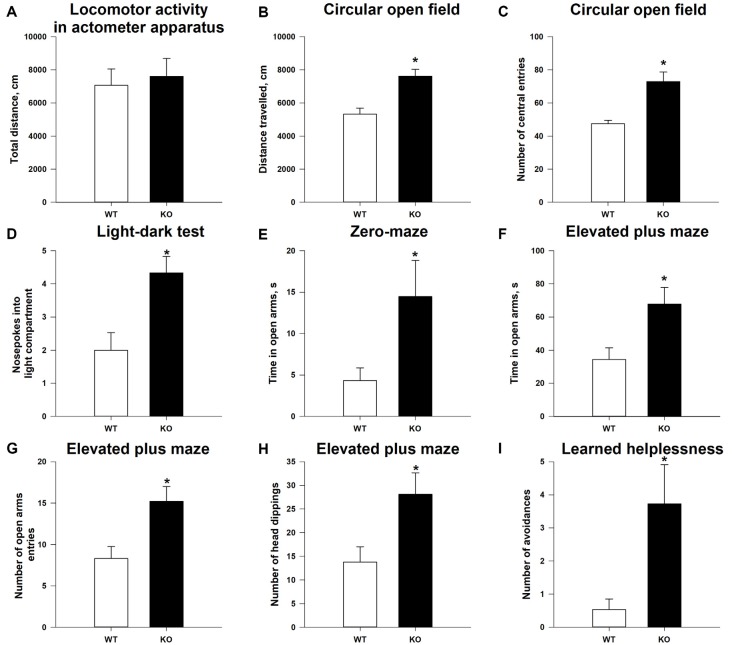
Reduced anxiety and affectivity of TAAR5-KO mice. **(A)** Minimally altered locomotor activity of TAAR5-KO mice in the locomotor activity boxes (measured for 60 min). Increased exploration **(B)** and number of central entries **(C)** of TAAR5-KO mice in the circular open field test. **(D)** Increased number of nose pokes into light compartment in the light–dark transition test. Increased time spent in open arms by TAAR5-KO mice in the elevated zero-maze test **(E)** and the elevated plus maze test **(F)**. Increased number of open arm entries **(G)** and head dippings **(H)** of TAAR5-KO mice in the elevated plus maze test. **(I)** Decreased affectivity of TAAR5-KO mice in the learned helplessness test (number of avoidances). *P < 0.05 (Student’s *t*-test or Mann–Whitney *U* test).

As shown in Supplementary Figure S1, the TAAR5-KO mice do not differ (*p* > 0.05) in general health, body weight, appearance of the fur and whiskers, and neurological reflexes and motor abilities (wire hang test: 57.9 + 2.10 s for WT vs. 59.7 + 0.33 s for TAAR5-KO) from WT littermates. The analysis of total distance traveled in the locomotor activity monitors also failed to reveal statistically significant differences between null mutants and WT mice (Figure 3A; *p* = 0.92). However, KO animals showed higher locomotor activity in the circular open field test that has a larger area for exploration: TAAR5-KO mice had higher distance traveled compared to WT animals (Figure 3B; *p* < 0.01). Furthermore, TAAR5-KO animals in the circular open field test showed increased number of the entrances to the central zone (*p* < 0.01), indicating decreased anxiety in mutant animals (Figure 3C).

### Lack of the TAAR5 Reduces Anxiety and Affectivity of Mice

Indeed, analysis of the anxiety state of TAAR5-KO mice in the light–dark box demonstrated that ablation of TAAR5 increased the number of nose pokes into the light compartment (Figure 3D; *p* < 0.05), but did not affect (*p* > 0.05) the time spent in the light compartment (87.2 ± 6.99 vs. 73.5 ± 10.67 s), the number of entries into the light compartment (7.1 ± 0.61 vs. 7.0 ± 0.52), and the latency of first entry into the light compartment (46.6 ± 12.16 vs. 41.0 ± 16.20 s). In addition, in the elevated zero-maze test, another common test used to assess the level of anxiety in mice, TAAR5-KO animals spent more time in the open arms (Figure 3E; *p* < 0.05), but showed similar latency entering into the open arms (92.8 ± 30.60 vs. 91.7 ± 34.14 s; *p* = 0.98). Furthermore, in the elevated plus maze test, TAAR5-KO animals also showed decreased anxiety, an increased number of entrances (Figure 3G; *p* < 0.05), an augmented total time spent in the open arms (Figure 3F; *p* < 0.01), and an increased number of head dippings (Figure 3H; *p* < 0.05). Ultimately, the learned helplessness test was performed to measure the affectivity of mutant and control mice. The TAAR5-KO mice performed significantly more avoidances than the WT animals (Figure 3I; *p* < 0.01), but *t*-test did not reveal significant differences in the total number of avoidances and escapes (12.0 ± 2.67 vs. 6.4 ± 2.41; *p* = 0.099) and the number of escapes (8.2 ± 1.82 vs. 5.8 ± 2.13; *p* = 0.58).

### Altered 5-HT Function in TAAR5-KO Mice

**Figure 4:**
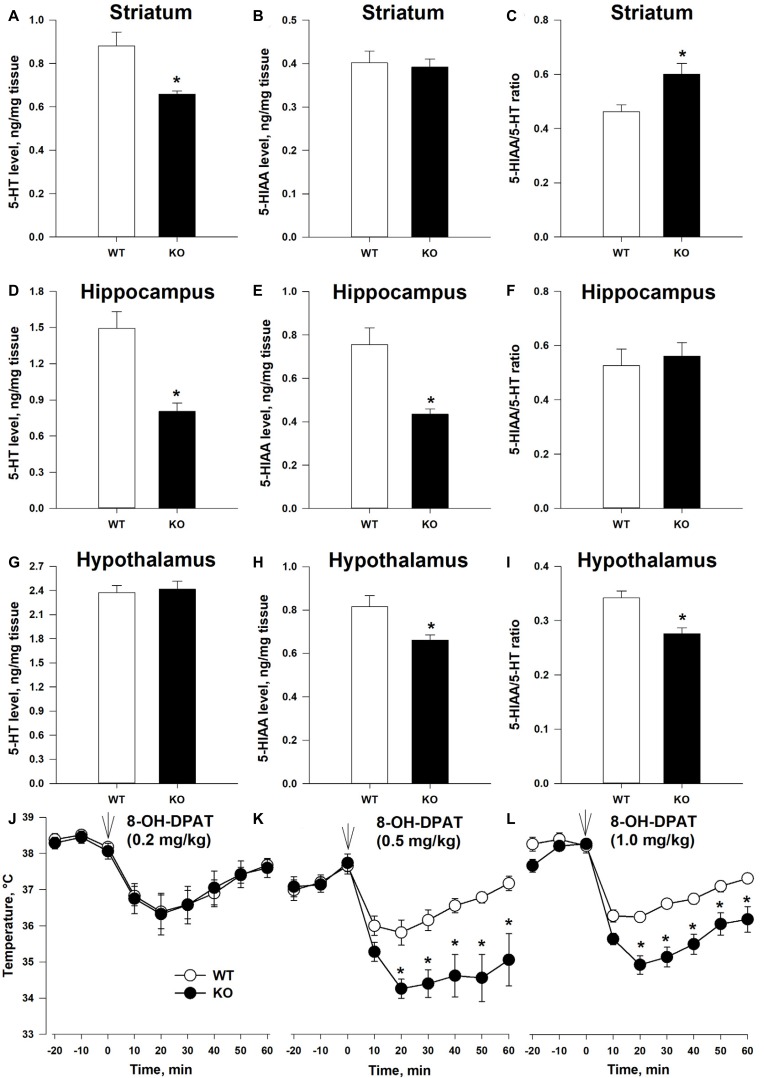
Altered 5-HT transmission in TAAR5-KO mice. **(A–C)** Tissue level of 5-HT, 5-HIAA, and turnover ratio 5-HIAA/5-HT in the striatum of WT and TAAR5-KO mice. **(D–F)** Tissue level of 5-HT, 5-HIAA, and turnover ratio 5-HIAA/5-HT in the hippocampus of WT and TAAR5-KO mice. **(G–I)** Tissue level of 5-HT, 5-HIAA, and turnover ratio 5-HIAA/5-HT in the hypothalamus of WT and TAAR5-KO mice. **(J–L)** Enhanced hypothermic effect of 5-HT1A agonist 8-hydroxy-2-(di-*n*-propilamino)tetralin (8-OH-DPAT; i.p.) in TAAR5-KO mice. *P < 0.05 [**(A–I)** Mann–Whitney *U* test; **(J–L)** two-way ANOVA followed by *post hoc* Bonferroni’s test for individual time point comparisons between genotypes].

Anxiety state and emotional behaviors in general are often connected to the function of the brain 5-HT system (Leonardo and Hen, 2006). To gain first insight on the possible connection between TAAR5 and the 5-HT system, we measured 5-HT and 5-HIAA tissue content in several brain structures (Figures 4A–I). 5-HT level was lower in TAAR5-KO mice in the striatum (Figure 4A; *p* < 0.005) and the hippocampus (Figure 4D; *p* < 0.005), while the level of 5-HIAA was lower in the hippocampus (Figure 4E; *p* < 0.005) and hypothalamus (Figure 4H; *p* < 0.01), resulting in alterations in 5-HT turnover rate expressed as ratio 5-HIAA/5-HT, in the striatum (Figure 4C; *p* < 0.005) and the hypothalamus (Figure 4I; *p* < 0.01**)**. At the same time, these parameters were not significantly altered in the frontal cortex of TAAR5-KO mice (5-HT: 1.23 ± 0.093 for WT vs. 1.08 ± 0.078 for TAAR5-KO, *p* = 0.37; 5-HIAA: 0.27 ± 0.029 for WT vs. 0.25 ± 0.018 for TAAR5-KO, *p* = 0.82; 5-HT turnover rate: 0.22 ± 0.017 for WT vs. 0.23 ± 0.009 for TAAR5-KO, *p* = 0.49).

The observed differences in 5-HT and 5-HIAA tissue content and 5-HT turnover rate in several brain areas of TAAR5-KO mice suggest that the lack of TAAR5 could affect the functional state of the brain 5-HT system. To check the hypothesis directly, we tested the effect of selective 5-HT1A agonist 8-hydroxy-2-(di-*n*-propilamino)tetralin (8-OH-DPAT) on centrally mediated thermoregulatory response (Gudelsky et al., 1986). The measurement of the temperature at basal state did not reveal differences between TAAR5-KO (35.9 ± 0.36°C) and WT (36.0 ± 0.26°C) animals (*p* = 0.7). However, as presented in Figures 4J–L, the hypothermic action of 8-OH-DPAT (0.5 and 1.0, but not 0.2 mg/kg, i.p.) was enhanced in the TAAR5-KO mice (the effect of factor “mutation”:*p* < 0.01, two-way ANOVA followed by *post hoc* Bonferroni’s test). To exclude a possible impact of lack of TAAR5 on general thermoregulation mechanism, the effect of selective muscarinic receptor agonist oxotremorine (0.05 mg/kg, i.p.) was tested in the mutant and control mice. The data obtained indicated that the TAAR5-KO and control mice demonstrate similar oxotremorine-induced hypothermic reaction (data not shown), the effect of factor “mutation” (*p* = 0.35, two-way ANOVA).

## Discussion

In this study, we observed that innate odor-detecting TAAR5 is expressed not only in the olfactory system but also in the major limbic brain areas processing olfactory information and is critically involved in the regulation of emotional behaviors. Furthermore, significant alteration in the function of brain serotonin was observed in the TAAR5-KO mice. Given a prominent role of serotonin system in the regulation of anxiety- and depression-related emotional behaviors (Leonardo and Hen, 2006), these data provide plausible mechanistic explanation for an altered emotional state of TAAR5-KO mice.

It is known that all the so-called human “olfactory” TAARs (with exception of TAAR1), including TAAR2, TAAR5, TAAR6, TAAR8, and TAAR9, are expressed in the olfactory epithelium and project to discrete glomeruli of the olfactory bulb (Johnson et al., 2012). In fact, the effect of TAAR5 deletion in the olfactory epithelium was carefully characterized, and loss of attraction to trimethylamine was found in TAAR5 knockout mice (Li et al., 2013; Liberles, 2015). Accordingly, using a LacZ reporter knock-in mouse, we could detect distinct groups of TAAR5-positive cells at the glomerular layer of the olfactory bulb, thereby validating this approach to map TAAR5 expression. However, unexpectedly, we detected TAAR5 expression also in deeper layers of the olfactory bulb (mitral/granular layers), which project to the brain limbic areas processing olfactory information. Indeed, TAAR5 expression was detected also in these areas (the anterior olfactory nucleus, the olfactory tubercle, the OFC, the amygdala, the hippocampus, the piriform cortex, the entorhinal cortex, the nucleus accumbens, the thalamic and hypothalamic nuclei) that are receiving olfactory input and critically involved in emotional behaviors. Interestingly, TAAR5 mRNA was found in the human amygdala when the human PNR, later renamed as TAAR5, was first cloned (Zeng et al., 1998). More recently, using *in situ* hybridization, Dinter and coworkers detected TAAR5 mRNA expression in the amygdala, as well as in the arcuate nucleus and ventromedial nucleus of hypothalamus of mice (Dinter et al., 2015). Thus, several lines of evidence in different species show that TAAR5 expression is not only restricted to the olfactory epithelium and the glomerular layer of olfactory bulb, but it is also present in the major limbic areas processing olfactory input and involved in emotional behaviors. It is notable that TAAR5 expression resembles the low and discrete pattern of expression of TAAR1 in monoaminergic nuclei (Lindemann et al., 2008). Certainly, *LacZ* reporter system has sensitivity limitations, thus possibly hindering TAAR5 detection in other brain areas. Further detailed investigations using immunohistochemical and other techniques are necessary to accurately map the pattern of brain expression of TAAR5 and to properly characterize somatic vs. neuropil expression profiles in the specific brain areas. It would be important also to evaluate expression pattern of TAAR5 in the peripheral organs.

The observed expression of TAAR5 in deeper layers of olfactory bulb and limbic brain areas processing olfactory information, alongside behavioral changes measured in the absence of TAAR5, provides a novel insight into mechanisms controlling innate emotional behaviors that are known to be affected in a number of psychiatric disorders. The importance of this pathway is underscored by the fact that, in rodents, the removal of the olfactory bulbs, i.e., olfactory bulbectomy, results in numerous alterations in the function of limbic brain regions, as well as behavioral changes, similar to those observed in depressed patients (Kelly et al., 1997). Because of the fact that the remarkable emotional behavioral deficits induced in olfactory bulbectomized animals are reversed after repeated administration of antidepressants, this model is commonly used to test the effectiveness of putative antidepressants (Morales-Medina et al., 2017). Indeed, in our study, we observed significant alterations in various measures of anxiety and affectivity of TAAR5-KO mice in a number of behavioral tests used to test putative anxiolytic and antidepressant drugs. Importantly, the lack of TAAR5 results in the anxiolytic/antidepressant-like phenotype, while recently identified putative nonselective TAAR5 receptor agonist 2-(alpha-naphthoyl) ethyltrimethylammonium iodide (alpha-NETA) increases cortical gamma-rhythm in rats (Belov et al., 2019), causes deficits in sensorimotor gating in rats (Aleksandrov et al., 2018a), and increases mismatch negativity (MMN)-like responses in rats and mice (Aleksandrov et al., 2018b, 2019) in a manner consistent with psychosis-related cognitive deficits described in humans and experimental animals (Light and Swerdlow, 2015).

Given the similarity in molecular and functional organization of other “olfactory” TAARs (Gainetdinov et al., 2018), we predict that other TAARs should be similarly expressed in limbic brain areas and involved in the regulation of emotional behaviors. However, since different TAARs are known to project to discrete glomeruli (Johnson et al., 2012), we speculate that they will be differently involved in transmission of olfactory information into the limbic “emotional” brain areas with specific patterns of brain expression and distinct contribution to emotional behaviors and monoamine system modulation. Thus, targeting TAARs in general could represent a novel multifaceted approach to the development of new classes of psychotropic drugs aimed at the control of emotional states that are known to be altered in many psychiatric disorders. With regard to TAAR5, it might be predicted that future development of selective TAAR5 antagonists could bring a new class of anxiolytic and/or antidepressant drugs. While the mechanism of such action requires further clarification, the general role of TAARs as modulators of classical brain monoaminergic neurotransmitters such as dopamine, serotonin, and norepinephrine should be considered. In fact, we observed significant alterations in the function of serotonin system in mice lacking TAAR5. Further studies involving detailed characterization of the serotonin system focused on serotonin synthesis rate, release, autoreceptor function, and monoamine oxidase (MAO) activity in different brain areas are necessary to fully understand the neurochemical mechanisms involved in these processes. Similarly, the effect of TAAR5 deletion on the status of dopamine and norepinephrine system should be carefully evaluated.

Taken together, we demonstrate here that TAAR5 (and likely TAARs in general) is not just “an olfactory receptor” involved in sensing innate odors but is also involved in the transmission of this information to emotional behaviors at the level of limbic brain areas likely through the modulation of the serotonin system. Thus, “olfactory” TAAR-mediated brain circuitry may represent a new type of neurotransmitter system involved in the transmission of innate odors into emotional behavioral responses. The potential impact of our findings to various fields of neuroscience, psychology, psychiatry, as well as novel principles of psychopharmacology and aromatherapy has not escaped our notice.

## Data Availability Statement

All datasets generated for this study are included in the article/Supplementary Material.

## Ethics Statement

The animal study was reviewed and approved by all procedures involving animals and their care were carried out in accordance with the guidelines established by the European Community Council (Directive 2010/63/EU of September 22, 2010) and were approved by the Italian Ministry of Health and Ethics Committee of St. Petersburg State University, St. Petersburg, Russia.

## Author Contributions

SE and RG designed the study, performed the experiments, analyzed the data, and wrote the manuscript. All the other authors performed the experiments, analyzed the data, and contributed to the writing of the manuscript. All authors revised and approved the final manuscript.

## Conflict of Interest

The authors declare that the research was conducted in the absence of any commercial or financial relationships that could be construed as a potential conflict of interest.

## Footnotes

^1^www.komp.org
