# Supplementary material for: Trace Amine-Associated Receptor 5 Provides Olfactory Input Into Limbic Brain Areas and Modulates Emotional Behaviors and Serotonin Transmission
Source: Front Mol Neurosci. 2020 Mar 5;13:18. doi: 10.3389/fnmol.2020.00018 (PMC7066256; doi:10.3389/fnmol.2020.00018)
Supplement: Supplementary file 1 [file Data_Sheet_1.pdf]

frontiers

Supplementary Material

Table S1. Number and gender of animals used in each experimental protocol.

Experimental protocol	WT		TAAR5-KO	
	Males	Females	Males	Females
LacZ histochemistry	5	6	8	6
Locomotor activity in actometer apparatus	6	5	5	6
Circular Open Field Test	9	-	9	-
Light-Dark Transition Test	9	-	6	-
Elevated Zero-Maze Test	7	2	10	3
Elevated Plus Maze Test	10	-	9	-
Learned Helplessness Test	11	6	12	10
HPLC measurements of the tissue content of serotonin and its metabolite	8	-	8	-
8-OH-DPAT-induced hypothermia test (0.2 mg/kg, i.p.)	4	4	5	3
8-OH-DPAT-induced hypothermia test (0.5 mg/kg, i.p.)	4	3	3	3
8-OH-DPAT-induced hypothermia test (1 mg/kg, i.p.)	3	3	4	4

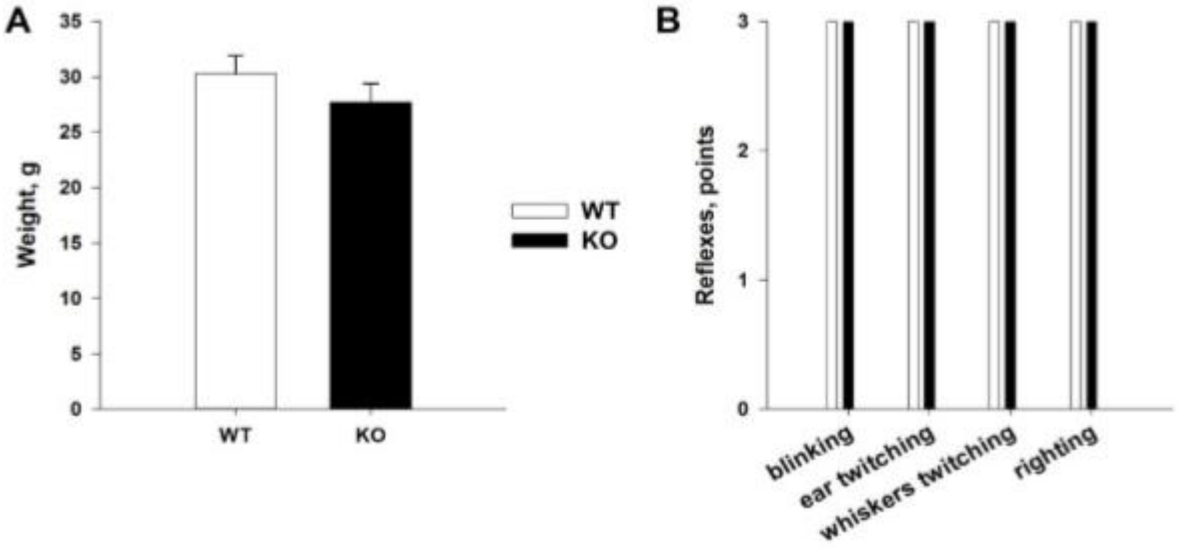

Figure S1. General behavioral profile of TAAR5-KO mice (both sexes). A. Weight of the animals (WT: n = 9; KO: n = 8). B. Sensory reflexes and motor abilities were evaluated in concordance with 3-point scale where 3 – norm; 2 – decreased state; 1 - absence. (WT: n = 13; KO: n = 10).
